# The association between early-life famine exposure and hypertension risk in adulthood and its interaction with dietary inflammatory index among Chinese adults

**DOI:** 10.3389/fnut.2026.1841432

**Published:** 2026-06-17

**Authors:** Jia Yin, Yiding Zhuang, Rui Qin, Zhixu Wang, Shanshan Geng, Ye Ding

**Affiliations:** 1 Department of Maternal, Child and Adolescent Health, School of Public Health, Nanjing Medical University, Nanjing, China; 2 Department of Laboratory Medicine, Affiliated Lianyungang Clinical College of Nantong University, Lianyungang, Jiangsu, China; 3 The Institute of Nutrition and Food Science, Nanjing Medical University, Nanjing, China; 4 Department of Nutrition and Food Safety, School of Public Health, Nanjing Medical University, Nanjing, China

**Keywords:** cohort study, dietary inflammatory index, early life, great Chinese famine, hypertension

## Abstract

**Background:**

Previous studies have shown that exposure to famine during early life is associated with long-term health outcomes. However, findings on the association between famine exposure and the risk of hypertension remain inconsistent. This study aimed to investigate the relationship between famine exposure at different early-life stages and the incidence of hypertension among Chinese adults, and further evaluate the interaction effect of Dietary Inflammatory Index (DII) on the risk of incident hypertension.

**Methods:**

Using data from the China Health and Nutrition Survey (2004–2015), a Chinese population-based cohort, participants were divided into three groups based on their birth year relative to the Great Chinese Famine: the fetal exposure group, the early childhood exposure group, and the non-exposure group. Cox regression was used to assess the association between famine exposure and incident hypertension. To further evaluate the interaction between famine exposure and DII on the risk of incident hypertension, DII was categorized into quartiles and incorporated into the analysis.

**Results:**

A total of 603 incident cases of hypertension were identified during the follow-up period. The early childhood exposure group exhibited a significantly higher risk of incident hypertension. Compared with the first DII quartile group, the third quartile group showed a 39% increased risk of incident hypertension. Additionally, a significant interaction was observed between DII and early childhood famine exposure in the fourth quartile group.

**Conclusions:**

Early childhood famine exposure showed a significant interaction with the DII, suggesting effect modification in the association between dietary inflammatory potential and adult hypertension. These findings highlight the potential value of targeted nutritional and dietary guidance for Chinese individuals with early-life nutritional adversity.

## 1 Introduction

Hypertension is one of the most prevalent non-communicable chronic diseases (NCDs) worldwide. According to the World Health Organization (WHO), the global number of hypertensive individuals had surged from 650 million in 1990 to 1.3 billion in 2019, with a prevalence of 33% among adults aged 30–79 years (1). Hypertension is also a significant contributor to the global disease burden, with elevated systolic blood pressure identified as the leading risk factor for mortality in the 2019 Global Burden of Disease Study (2). The epidemiological landscape of hypertension in China remains concerning. Studies indicated that the age-standardized prevalence of hypertension among Chinese adults aged 18–69 years rose from 20.8% in 2004 to 29.6% in 2010, before declining to 24.7% in 2018 (3). Although this downward trend suggests potential improvements in population-level interventions, public awareness, and treatment rates among Chinese adults remain suboptimal. Notably, emerging evidence suggests that younger age at hypertension onset is associated with a stronger correlation with all-cause cardiovascular mortality (4). Thus, identifying and mitigating risk factors for hypertension is crucial for establishing a three-tier prevention framework. Beyond conventional adult lifestyle risk factors, increasing attention has been paid to early-life exposures that may shape long-term cardiovascular susceptibility and contribute to hypertension risk in later life (5, 6).

In 1990, Barker proposed the Developmental Origins of Health and Disease (DOHaD) hypothesis, positing that adverse early-life exposures significantly elevate the risk of NCDs in adulthood (7). Numerous retrospective observational studies based on extreme events have demonstrated that early-life nutritional deprivation is associated with cardiovascular diseases, impaired glucose tolerance, cancer, osteoporosis, and mental disorders in adulthood (8–10). Famine exposure represents an extreme form of early-life nutritional adversity and has therefore been widely used as a natural experiment to investigate the developmental origins of adult chronic diseases (11, 12). Regarding hypertension, several studies have explored the relationship between famine exposure in early life and hypertension risk, but these studies are mainly cross-sectional designs (13). Cohort-based evidence remains limited and is largely restricted to specific regions, with a lack of large-scale studies involving geographically diverse populations. In limited cohort-based evidence, a cohort study by Hult et al. (14) revealed that, compared to individuals who did not experience famine, individuals exposed to famine during the fetal and infancy stages faced a 2.87-fold increased risk (95% CI: 1.90–4.34) of developing hypertension in adulthood. For those exposed during childhood, the risk increased by 1.77-fold (95% CI: 1.17–2.86). Despite these studies, the association between famine exposure and hypertension remains inconclusive. Zhao et al. (15) found that individuals exposed to famine in early life had a lower risk of developing hypertension in adulthood. Therefore, large-scale longitudinal studies with geographically diverse populations are needed to further clarify the association between early-life famine exposure and hypertension in adulthood.

Studies have suggested that early-life nutritional status may be associated with a higher risk of hypertension, potentially through its influence on dietary behaviors in adulthood. Individuals exposed to early-life malnutrition often exhibit an increased preference for high-energy-dense foods (e.g., high-sugar, high-fat diets) in adulthood, which may significantly contribute to their elevated risk of developing hypertension and other NCDs (16). Such energy-dense dietary patterns have often been linked to higher dietary inflammatory potential. The Dietary Inflammatory Index (DII), as a quantitative scoring index that assesses the overall inflammatory potential of a diet at the individual level, integrates up to 45 dietary and nutritional components to summarize where an individual's diet lies on a continuum from more anti-inflammatory to more pro-inflammatory. Before the DII was developed, most dietary indices used in epidemiologic research broadly fell into three categories (17): indices based on dietary recommendations, indices reflecting adherence to a particular dietary tradition or cuisine, and data-driven indices derived within specific study populations. These approaches summarize diet from different perspectives, whereas the DII provides a complementary, literature-derived dimension focused on dietary inflammatory potential that can be applied across diverse dietary contexts. Currently, multiple studies (18, 19) have reported associations between this index and NCD outcomes, supporting the hypothesis that diet-related inflammatory potential may be relevant to NCD risk. However, current evidence on the association between early-life famine exposure and adult dietary characteristics, including dietary inflammatory potential, remains relatively limited, and existing studies show inconsistent findings (20, 21). Therefore, further research is warranted to examine whether dietary inflammatory potential modifies the association between early-life famine exposure and adult hypertension risk, which may help inform life-course prevention strategies for hypertension.

The Great Chinese Famine (1959–1961) was one of the largest famines in human history, causing widespread nutritional deprivation across China (22, 23). As a large-scale historical event occurring within a clearly defined time window, it provides a unique natural experimental setting for examining the long-term health consequences of early-life undernutrition. Individuals born around the famine period have now reached middle and older adulthood, a life stage during which hypertension becomes increasingly common. Therefore, based on this historical event, investigating the long-term association between early-life famine exposure and incident hypertension is important for understanding life-course determinants of cardiovascular risk and for identifying Chinese adults who may benefit from targeted hypertension prevention.

Using data from the China Health and Nutrition Survey (CHNS), this study aimed to examine the association between early-life famine exposure and incident hypertension among middle-aged and older Chinese adults, and to evaluate whether dietary inflammatory potential modified this association. By integrating early-life famine exposure, repeated dietary assessments, and longitudinal follow-up for hypertension, this study may help identify high-risk populations and inform targeted prevention strategies in China.

## 2 Methods

### 2.1 Study population

This study utilized data from CHNS, an ongoing, prospective, open-cohort study employing multistage, stratified cluster sampling. Initiated in 1989, the CHNS has completed 10 survey rounds across 15 provinces/autonomous regions/municipalities in China, encompassing approximately 388 communities, 11,130 households, and over 40,000 participants. It systematically evaluated the dynamic changes in demographics, socioeconomic status, nutrition and health at the individual and household levels. It was approved by the institutional ethics committee and all participants provided written informed consent. Detailed CHNS protocols were described in prior publications (24).

**Figure 1:**
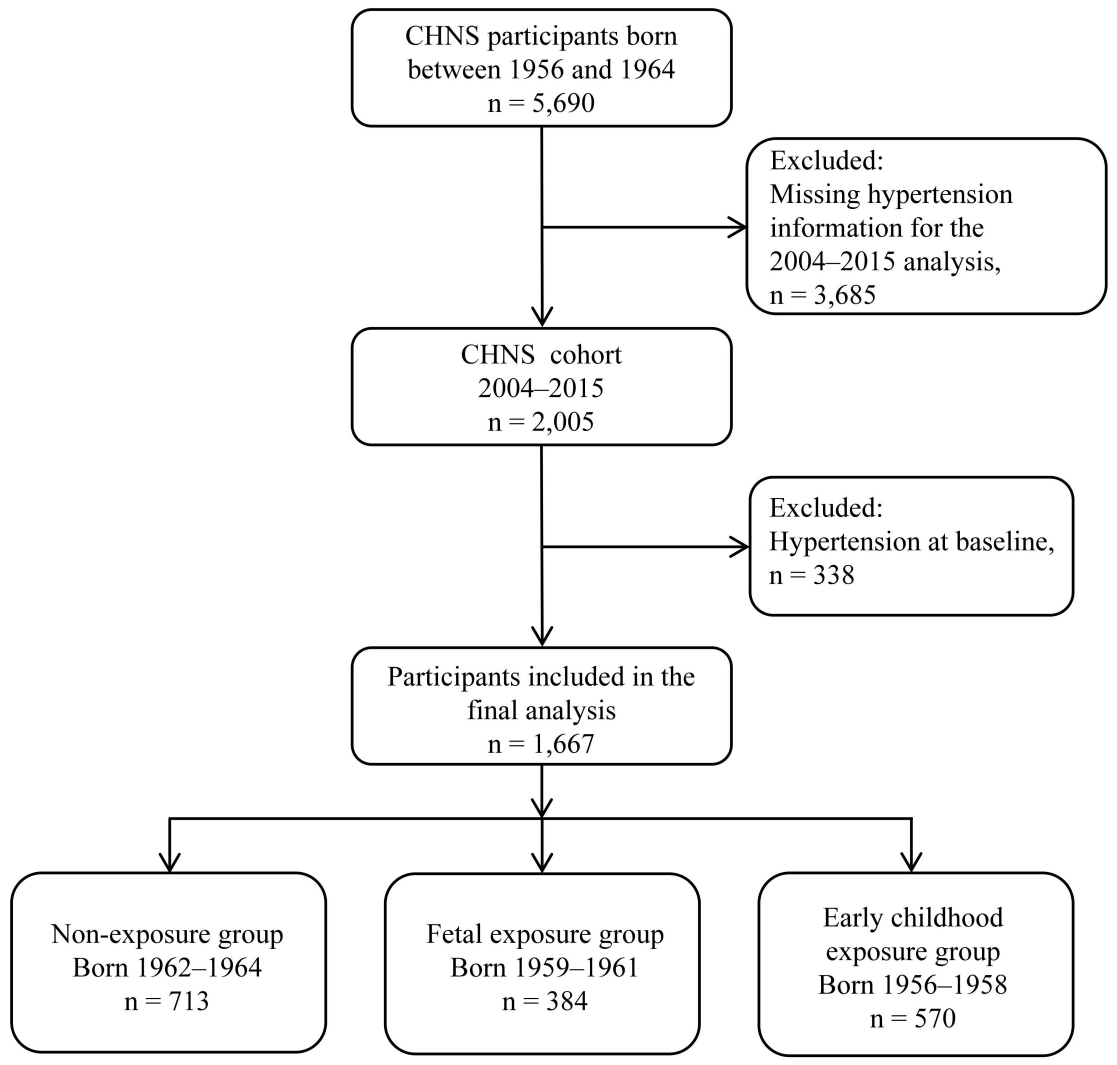
Flow chart of participant selection.

In the follow-up of the year 2000 and earlier, dietary survey data were mainly recorded and calculated based on the “Chinese Food Composition Table (1991 edition)”, and the early sample size was relatively small. To ensure data consistency and comparability, 2004 was established as the baseline time point and 2015 as the follow-up endpoint in this study. From the initial 36,996 individuals in the population-based cohort, 5,690 participants born between 1956 and 1964 were identified. After excluding those with missing hypertension information (*n* = 3,685) and those with hypertension at baseline (*n* = 338), a total of 1,667 Chinese individuals were included in the final analytic sample. The detailed participant selection process is shown in Figure 1.

### 2.2 Definition of exposure and control

**Figure 2:**
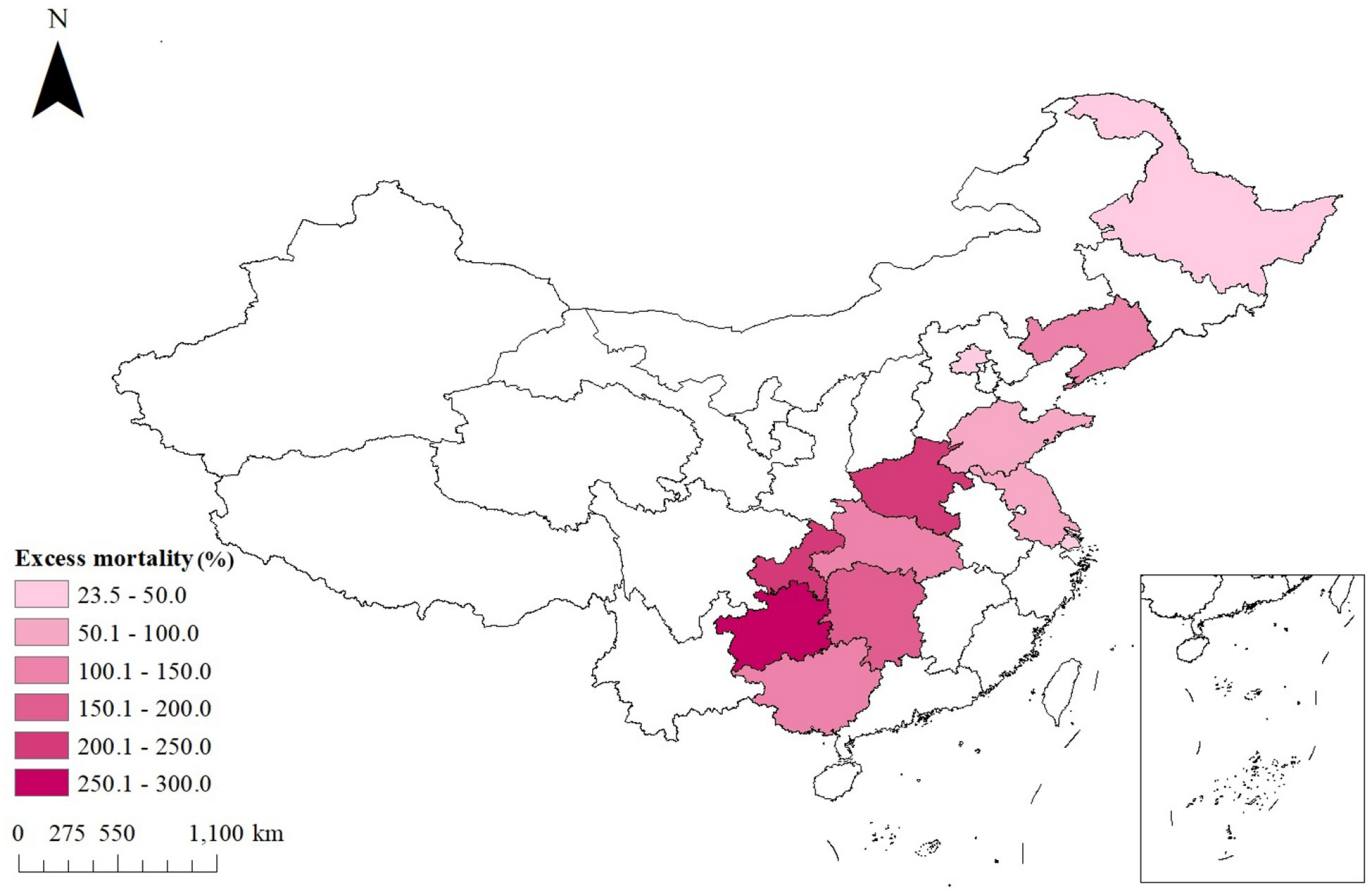
Distribution of CHNS survey sites and provincial excess mortality in China. Maps are derived from the National Platform for Common GeoSpatial Information Services / Tianditu Service map approval number: GS (2024) 0650, Ministry of Natural Resources of the People's Republic of China. The base map boundaries were not modified. The figure was produced by the authors using QGIS.

The Great Chinese Famine (1959–1961) affected all regions of China, but the severity of the famine varied regionally due to disparities in climate, population density, and the implementation of policies related to food shortages. Following prior studies, participants were stratified by birth year (25, 26): fetal exposure group (born 1959–1961, exposed during the fetal period), early childhood exposure group (born 1956–1958, exposed during early childhood), and non-exposure group (born 1962–1964, no prenatal or postnatal famine exposure). This study used the excess mortality rate (EMR) as a regional-level proxy to assess famine severity. The EMR is defined as the percentage change in the highest mortality rate during 1959–1962 relative to the average mortality rate in 1956–1958 (27). Based on previous research, an EMR exceeding 50% is classified as a severe famine region, while an EMR below 50% is considered a less severe region (28, 29). According to the literature (30), the survey sites in China and the distribution of excess mortality rates are presented in Figure 2.

### 2.3 Determination of hypertension

Hypertension was defined as meeting any of the following criteria (31): (1) mean systolic blood pressure (SBP) ≥140 mmHg, (2) mean diastolic blood pressure (DBP) ≥90 mmHg, (3) self-reported physician diagnosis, or (4) self-reported antihypertensive medication use. SBP and DBP were calculated as the average of three measurements taken on the same day. On this basis, incident hypertension was defined as the first occurrence of hypertension during follow-up among participants without hypertension at baseline in 2004.

### 2.4 Dietary assessment

All trained investigators conducted face-to-face dietary surveys using a combined approach of individual-level 24-h dietary recall over three consecutive days and household-level food weighing records during the same 3-day period (24). For individual dietary surveys, each family member reported all foods consumed during the previous 24 h, regardless of where the foods were consumed. Household food consumption (cooking oil and seasonings) was determined by measuring inventory changes combined with weighing. Dietary data were calculated using the Chinese Food Composition Table (6th Edition) to estimate energy and nutrient intakes (32). Following the methodology of Du et al. (33), intake from household-level condiments was apportioned to individual members based on relative energy consumption, and participants' average individual-level sodium and potassium intake from 2004 to 2011 was subsequently calculated for use in sensitivity analysis.

For the present study, dietary assessment data were available in four CHNS survey waves, namely 2004, 2006, 2009, and 2011, and these waves were used to derive the DII. To capture longer-term dietary inflammatory exposure and reduce random within-person variation, the primary analysis used the average DII across all available waves from 2004 to 2011.

### 2.5 Calculation of dietary inflammatory index

Based on the available dietary data, the following 23 dietary and nutritional components were used to calculate DII: energy, carbohydrates, dietary fiber, protein, total fat, saturated fatty acids, monounsaturated fatty acids, polyunsaturated fatty acids, cholesterol, vitamin A, β-carotene, vitamin B_1_, vitamin B_2_, vitamin B_6_, vitamin B_12_, niacin, folate, vitamin C, iron, magnesium, zinc, selenium, and alcohol. At present, in various studies exploring the association between DII and diseases, the number of dietary and nutritional components used is predominantly between 20 and 30 (34–36). Previous studies have shown that DII still has the ability to predict inflammation when the dietary and nutritional components are fewer than 45 complete parameters (37, 38).

The specific formula and important parameters are as follows (39), and the overall DII score for each participant was derived by summing all adjusted DII scores. The DII is interpreted such that more negative values reflect greater anti-inflammatory dietary potential, while more positive values indicate greater pro-inflammatory potential. Based on the full set of 45 food parameters, the theoretical range of the DII has been reported as approximately −8.87 to +7.98. In most epidemiologic studies, where the DII is typically derived from around 25 dietary parameters, the observed range is commonly narrower, often approximately −5.5 to +5.5 (17).


$$\begin{array}{lll} DII_{\;i\;} & = & \frac{Daily\;intake\;-Global\;mean\;intake}{Global\;intake\;SD} \end{array}$$ (1)

$$\begin{array}{lll} {DII}_{i\;adjusted} & = & 2\;\times \;\Phi \left(DII_{i}\right)-1 \end{array}$$ (2)

$$\begin{array}{lll} DII & = & \;\overset{n}{\underset{i=1}{\sum }}DII_{i\;adjusted}\;\times \;{inflammatory\;effect\;score}_{i} \end{array}$$ (3)

DII *i*: DII of a particular dietary or nutritional component.

Φ (): Used to obtain the percentile value.

DII_*i*_ adjusted: Adjusted DII Score of a particular dietary or nutritional component after standardization and symmetrization.

DII: DII score at the individual level.

### 2.6 Covariate assessment

Relevant information was obtained through face-to-face questioning and standardized calculation by trained and qualified investigators. The covariates considered included demographic characteristics, lifestyle behaviors, and dietary intake variables. Demographic covariates included age, gender, residence area, education level, and BMI. Age and BMI were treated as continuous variables. BMI was calculated as weight in kilograms divided by height in meters squared. Gender was categorized as male or female. Residence area was categorized as urban or rural. Education level was categorized as ≤ 9 years or >9 years. Lifestyle covariates included smoking status, alcohol consumption, and physical activity level. Smoking status was assessed through the questionnaire item “Have you ever smoked cigarettes? (including hand-rolled, machine-rolled, and pipe tobacco)”, and alcohol consumption was evaluated using the questionnaire item “Did you drink beer, liquor, or other alcoholic beverages last year?”. Physical activity was categorized as either low or moderate-to-high intensity. The mean daily energy intake, calculated from the 3-day dietary assessment, was treated as a continuous variable. In the regression models, the reference categories were male for gender, urban residence for residence area, ≤ 9 years for education level, never-smoker for smoking status, never-drinker for alcohol consumption, and low physical activity for physical activity level. For famine exposure, the non-exposure group was used as the reference category. DII quartiles were modeled as categorical variables, with the first quartile used as the reference group.

### 2.7 Statistical analysis

Since the continuous variables did not follow a normal distribution after normality testing, the data were presented as *P*_50_ (*P*_25_, *P*_75_) with the Kruskal–Wallis test for intergroup comparison. Categorical variables were described as *n* (%), and group differences were evaluated using the chi-square test.

Using famine exposure at different stages of early life as the independent variables, we performed Cox proportional hazards models for incident hypertension, with the non-exposure group as the reference group. Following similar studies (13, 40, 41), three sequential models were built: Model 1 adjusted for gender only; Model 2 expanded upon Model 1 by additionally adjusting for other demographic characteristics (residence area, education level, and BMI); and Model 3 further incorporated lifestyle behaviors (smoking status, alcohol consumption, physical activity level, and mean daily intakes of energy) into Model 2 for comprehensive adjustment. As a sensitivity analysis, missing covariates were imputed using multiple imputation by chained equations with the mice package in R. Five imputed datasets were generated with 10 iterations using predictive mean matching. Estimates were pooled using Rubin's rules. Building upon this multiple-imputation sensitivity analysis, we further conducted another sensitivity analysis. We incorporated participants' average individual-level sodium and potassium intake as continuous covariates into Model 3, in order to evaluate whether the observed associations were robust to adjustment for these key dietary determinants of blood pressure.

Additionally, DII was categorized into quartiles to construct interaction terms with famine exposure to examine whether the association between DII and incident hypertension differed across early-life famine exposure groups. Finally, further exploration was conducted on whether exposure to famine affected the relationship between DII and the incidence of hypertension. Given that dietary behaviors may change following a hypertension diagnosis, we conducted a sensitivity analysis to reduce potential reverse causality. For participants who developed incident hypertension during follow-up, DII was not updated after the event, and only dietary assessments collected prior to the first event were used to calculate mean DII.

All analyses were performed using R software (version 4.4.1), with two-sided *P*-values <0.05 considered statistically significant. QGIS was used for map visualization.

## 3 Results

### 3.1 Baseline characteristics of participants

The study included a total of 1,667 participants, including 769 males (46.1%) and 898 females (53.9%), with a median age of 43.00 years. Among them, 603 individuals (36.2%) developed incident hypertension during follow-up (2004–2015), and 1,064 participants (63.8%) remained hypertension-free throughout the period.

As shown in Table 1, participants were stratified into three groups: non-exposure group (*n* = 713), fetal exposure group (*n* = 384), and early childhood exposure group (*n* = 570), with median ages of 41.00, 44.00, and 47.00 years, respectively. Significant intergroup differences (*P* < 0.05) were observed in age and education level. No significant differences were found among the groups in gender, residence area, BMI, smoking status, alcohol consumption, physical activity level, or daily energy, protein, fat, carbohydrate, sodium and potassium intake. No statistically significant difference was observed in the incidence of hypertension across different early-life famine exposure groups.

**Table 1:** Baseline characteristics of populations exposed to famine at different stages of early life [*P*_50_ (*P*_25_, *P*_75_)].

Variables	Total	Non-exposure group	Fetal exposure group	Early childhood exposure group	*P*
*N*	1,667	713	384	570	
Demographic characteristics					
Age (years)	43.00 (41.00, 46.00)	41.00 (40.00, 41.00)	44.00 (43.00, 45.00)	47.00 (46.00, 48.00)	<0.001
Gender [*n* (%)]					
Male	769 (46.1)	333 (46.7)	178 (46.4)	258 (45.3)	0.872
Female	898 (53.9)	380 (53.3)	206 (53.6)	312 (54.7)	
Residence area [*n* (%)]					
Urban	549 (32.9)	232 (32.5)	141 (36.7)	176 (30.9)	0.163
Rural	1,118 (67.1)	481 (67.5)	243 (63.3)	394 (69.1)	
Famine severity [*n* (%)]					
Less severe	157 (9.4)	62 (8.7)	43 (11.2)	52 (9.1)	0.383
Severe	1,510 (90.6)	651 (91.3)	341 (88.8)	518 (90.9)	
Education level [*n* (%)]					
≤ 9 years	909 (54.5)	423 (59.3)	198 (51.6)	288 (50.5)	<0.001
>9 years	481 (28.9)	213 (29.9)	126 (32.8)	142 (24.9)	
Missing	277 (16.6)	77 (10.8)	60 (15.6)	140 (24.6)	
BMI (kg/m^2^)	23.0 (21.0, 25.0)	22.9 (21.2, 24.9)	23.0 (21.1, 25.2)	23.0 (20.9, 24.9)	0.668
Lifestyle behaviors					
Smoking status [*n* (%)]					
Never-smoker	1,090 (65.4)	467 (65.5)	239 (62.2)	384 (67.4)	0.162
Current/former smoker	573 (34.4)	246 (34.5)	144 (37.5)	183 (32.1)	
Missing	4 (0.2)	0 (0.0)	1 (0.3)	3 (0.5)	
Alcohol consumption [*n* (%)]					
Never-drinker	1,042 (62.5)	421 (59.0)	256 (66.7)	365 (64.0)	0.088
Current/former-drinker	619 (37.1)	288 (40.4)	127 (33.1)	204 (35.8)	
Missing	6 (0.4)	4 (0.6)	1 (0.3)	1 (0.2)	
Physical activity level [*n* (%)]					
Low	928 (55.7)	392 (55.0)	221 (57.6)	315 (55.3)	0.806
Moderate-to-high	695 (41.7)	302 (42.4)	151 (39.3)	242 (42.5)	
Missing	44 (2.6)	19 (2.7)	12 (3.1)	13 (2.3)	
Dietary intake					
Energy intake (kcal/d)	2,301.5 (1,827.2, 2,738.4)	2,321.3 (1,829.2, 2,746.4)	2,307.2 (1,859.7, 2,730.2)	2,269.8 (1,818.7, 2,718.1)	0.698
Carbohydrate intake (g/d)	319.5 (253.0, 401.3)	327.2 (256.1, 411.0)	318.7 (252.8, 399.0)	309.8 (250.5, 394.8)	0.194
Fat intake (g/d)	68.5 (46.7, 94.8)	67.2 (46.2, 91.4)	71.1 (49.1, 101.6)	67.5 (46.4, 95.5)	0.257
Protein intake (g/d)	67.7 (53.3, 82.7)	69.2 (54.2, 83.5)	66.7 (53.0, 82.4)	66.5 (53.1, 82.2)	0.366
Sodium (g/d)	4.81 (3.85, 6.19)	4.76 (3.71, 5.95)	4.95 (3.85, 6.30)	4.80 (3.99, 6.36)	0.086
Potassium (g/d)	2.53 (2.15, 2.98)	2.55 (2.15, 2.97)	2.48 (2.15, 2.95)	2.54 (2.14, 3.01)	0.603
Incident hypertension [*n* (%)]		
No	1,064 (63.8)	474 (66.5)	246 (64.1)	344 (60.4)	0.076
Yes	603 (36.2)	239 (33.5)	138 (35.9)	226 (39.6)	

BMI, body mass index; Data are expressed as *P*_50_ (*P*_25_, *P*_75_) or *n* (%); Intergroup comparisons were performed using the chi-square test or the nonparametric Kruskal–Wallis test. Missing values for continuous covariates included in the Cox regression models were as follows: BMI, *n* = 116 (7.0%); mean daily energy intake over 3 days, *n* = 25 (1.5%).

### 3.2 The association between early-life exposure to famine and the risk of hypertension in adulthood

As shown in Table 2, using the non-exposure group as reference, Cox proportional hazards regression models demonstrated that early childhood exposure to famine was significantly associated with an increased risk of incident hypertension in Model 1 (HR = 1.22, 95% CI: 1.02–1.46) and Model 3 (HR = 1.24, 95% CI: 1.02–1.53). No significant association was found between fetal exposure and the risk of incident hypertension.

**Table 2:** Cox regression analysis of famine exposure during different early-life stages and risk of incident hypertension.

Exposure group	Incidence (%)	Model 1		Model 2		Model 3	
		HR (95% CI)	*P*	HR (95% CI)	*P*	HR (95% CI)	*P*
Main analysis							
Non-exposure group	33.5	Ref.		Ref.		Ref.	
Fetal exposure group	35.9	1.07 (0.87–1.33)	0.503	0.97 (0.76–1.24)	0.818	0.96 (0.75–1.22)	0.731
15.5-7.4,-13.5175.3mmEarly childhood exposure group	39.6	1.22 (1.02–1.46)^*^	0.032^*^	1.21 (0.98–1.50)	0.078	1.24 (1.02–1.53)^*^	0.050^*^
Sensitivity analysis							
Non-exposure group	33.5	Ref.		Ref.		Ref.	
Fetal exposure group	35.9	1.07 (0.87–1.32)	0.504	1.07 (0.87–1.32)	0.510	1.06 (0.86–1.31)	0.567
Early childhood exposure group	39.6	1.22 (1.02–1.46)^*^	0.032^*^	1.21 (1.02–1.45)	0.039^*^	1.22 (1.02–1.46)^*^	0.034^*^

CI, confidence interval; Model 1 included gender as an adjustment variable; Model 2 included gender, residence area, educational level, and BMI as adjustment variables; Model 3 included gender, residence area, educational level, BMI, smoking status, alcohol consumption, physical activity level, and mean daily intakes of energy over 3 days as adjustment variables; ^*^Compared with the non-exposure group, *P* < 0.05.

The results of the sensitivity analysis further validated the robustness of the findings above. For incident hypertension, early childhood famine exposure remained significantly associated with incident hypertension across all three models (Model 1: HR = 1.22, 95% CI: 1.02–1.46; Model 2: HR = 1.21, 95% CI: 1.02–1.45; Model 3: HR = 1.22, 95% CI: 1.02–1.46). No statistically significant association was observed between fetal famine exposure and the risk of incident hypertension in either the main analysis or the sensitivity analysis. In Supplementary Table S1, sodium and potassium intake were further adjusted for in Model 3, and the results remained robust.

### 3.3 Stratified analysis of famine exposure at different stages of early life and the risk of hypertension

The results of the stratified analysis showed significant heterogeneity in the association between exposure to famine at different early-life stages and the risk of incident hypertension. No significant associations were observed for either fetal exposure or early childhood exposure in the urban–rural strata or across the ≤ 9- and >9-year education strata. In the imputed alcohol consumption–stratified analysis, current/former drinkers had a significantly increased risk of hypertension after early childhood exposure (HR = 1.40, 95% CI: 1.05–1.86), whereas no significant association was observed among never drinkers. In the famine severity–stratified analysis, early childhood exposure was associated with a significantly increased risk of incident hypertension in severely affected areas (HR = 1.28, 95% CI: 1.04–1.58), whereas no significant association was found in less severely affected areas. The complete risk profiles are shown in Table 3.

**Table 3:** Stratified analysis of the association between famine exposure during different early-life stages and risk of incident hypertension.

Analysis	Fetal exposure group vs. Non-exposure group		Early childhood exposure group vs. Non-exposure group	
	HR (95% CI)	*P*	HR (95% CI)	*P*
Residence-stratified				
Urban	1.13 (0.73–1.75)	0.583	1.30 (0.86–1.97)	0.213
Rural	0.89 (0.66–1.20)	0.453	1.18 (0.92–1.52)	0.185
Education-stratified				
≤ 9 years	1.14 (0.85–1.54)	0.374	1.23 (0.94–1.60)	0.130
>9 years	0.67 (0.43–1.04)	0.071	1.27 (0.87–1.85)	0.213
Education-stratified (imputed)				
≤ 9 years	1.22 (0.94–1.57)	0.128	1.24 (0.99–1.55)	0.059
>9 years	0.81 (0.55–1.18)	0.270	1.21 (0.87–1.67)	0.260
Alcohol consumption-stratified				
Never-drinker	1.11 (0.81–1.52)	0.507	1.22 (0.92–1.63)	0.167
Current/former-drinker	0.78 (0.52–1.17)	0.229	1.23 (0.89–1.70)	0.201
Alcohol consumption-stratified (imputed)				
Never-drinker	1.18 (0.91–1.53)	0.212	1.14 (0.90–1.45)	0.263
Current/former-drinker	0.90 (0.63–1.30)	0.581	1.40 (1.05–1.86)^*^	0.021^*^
Famine severity				
Less severe	1.24 (0.55–2.78)	0.605	1.18 (0.53–2.64)	0.685
Severe	0.99 (0.78–1.27)	0.949	1.28 (1.04–1.58)	0.022^*^

CI, confidence interval; the analysis of incident hypertension used the Cox proportional hazards regression model; The model included gender, residence area, educational level, BMI, smoking status, alcohol consumption, physical activity level, and mean daily intakes of energy over 3 days, except for the corresponding stratification variable in each subgroup analysis; ^*^Compared with non-exposure group, *P* < 0.05.

### 3.4 DII status in different years among groups exposed to famine at varying stages of early life

The median DII scores in the total population were −1.33, −1.01, −1.25, and 1.08 for the years 2004, 2006, 2009, and 2011, respectively. When comparing DII scores between incident hypertension cases and non-cases within the same exposure groups, a statistically significant difference was observed only in the non-exposure group for the year 2004, indicating significantly higher DII scores among incident hypertension cases compared to non-cases. No statistically significant differences in DII scores were observed between cases and non-cases in either the fetal exposure group or the early childhood exposure group across all survey years. Specific characteristics are shown in Table 4. The overall median of the mean DII values across these four surveys was −0.67. The DII scores for each survey year and the overall DII level from 2004 to 2011 among the three groups were summarized in Supplementary Table S2 as *P*_50_ (*P*_25_, *P*_75_), with a significant difference observed between the early childhood exposure and the non-exposure groups in 2006 (Benjamini–Hochberg–adjusted *P* < 0.05).

**Table 4:** Dietary inflammatory index by year at different early-life stages of famine exposure and incident hypertension stratification [*P*_50_ (*P*_25_, *P*_75_)].

DII assessment	Total	Non-exposure group		Fetal exposure group		Early childhood exposure group	
		Cases	Non-cases	Cases	Non-cases	Cases	Non-cases
***N***	1,667	239	474	138	246	226	344
**DII 2004**	−1.33 (−2.72, 0.27)	−1.15 (−2.51, 0.72)^**^	−1.64 (−3.08, 0.06)^**^	−1.07 (−2.34, 0.37)	−1.72 (−2.56, 0.31)	−1.25 (−2.69, 0.33)	−1.35 (−2.98, 0.00)
**DII 2006**	−1.01 (−2.58, 0.62)	−1.04 (−2.36, 0.46)	−0.94 (−2.67, 0.37)	−1.22 (−2.71, 0.91)	−1.15 (−2.47, 0.49)	−0.82 (-2.32, 0.75)	−0.68 (-2.04, 0.95)
**DII 2009**	−1.25 (−2.61, 0.27)	−1.05 (−2.47, 0.48)	−1.13 (−2.53, 0.37)	−1.28 (−3.01, 0.36)	−1.19 (−2.56, 0.25)	−1.32 (-2.96, 0.18)	−1.10 (−2.36, 0.26)
**DII 2011**	1.08 (−0.19, 2.20)	0.93 (−0.21, 2.43)	0.73 (−0.56, 1.84)	1.07 (−0.25, 2.14)	1.24 (0.01, 2.13)	1.01 (−0.28, 2.08)	1.35 (−0.06, 2.32)
**DII 2004–2011**	−0.67 (−1.73, 0.36)	−0.64 (−1.72, 0.50)	−0.84 (−1.75, 0.15)	−0.72 (−1.84, 0.40)	−0.52 (−1.38, 0.47)	−0.69 (−1.80, 0.40)	−0.48 (−1.57, 0.52)

DII, dietary inflammatory index; DII 2004, DII 2006, DII 2009, and DII 2011 represent the Dietary Inflammatory Index for the years 2004, 2006, 2009, and 2011, respectively; The Dietary Inflammatory Index is calculated based on the food nutrient components and nutrients derived from dietary surveys; DII 2004–2011 represents the average Dietary Inflammatory Index from 2004 to 2011; Intergroup comparisons were performed using the Mann–Whitney *U* test; ^**^Compared with study participants without incident hypertension, *P* < 0.01.

### 3.5 Effect modification by early-life famine exposure in the association between DII and incident hypertension

**Figure 3:**
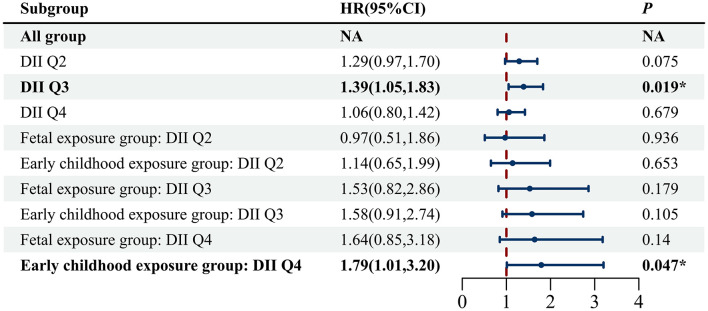
Interaction between exposure to early-life famine and Dietary Inflammatory Index on the incidence of hypertension. DII, dietary inflammatory index; the Dietary Inflammatory Index is calculated based on food nutrient components and nutrients derived from dietary surveys; DII Q2, DII Q3, and DII Q4 represent the second, third, and fourth quartiles (Q2, Q3, Q4) of the Dietary Inflammatory Index, respectively, with Q1 as the reference group; CI: confidence interval; *Compared with the reference group, *P* < 0.05; Adjusted for age, residence area, gender, educational level, BMI, smoking status, alcohol consumption, physical activity level, and mean daily intakes of energy over 3 days.

**Figure 4:**
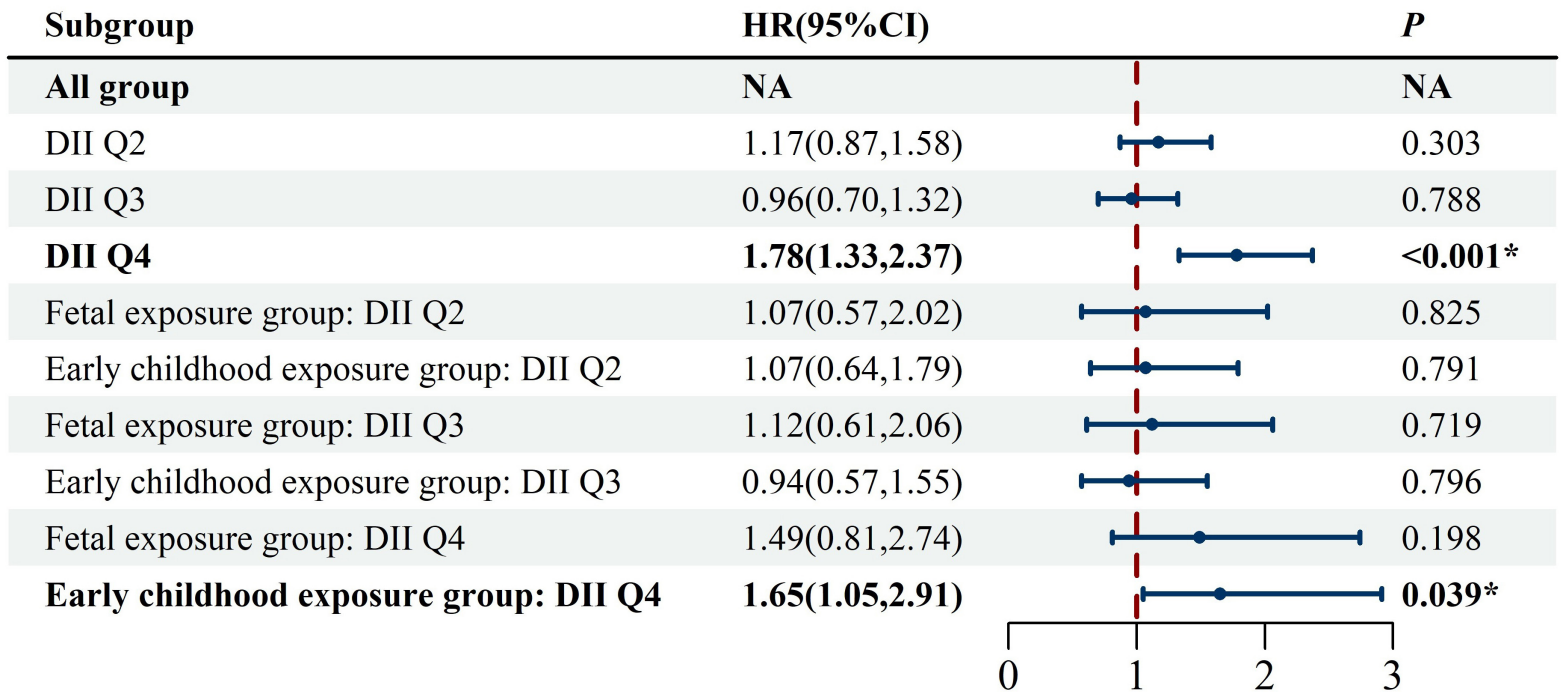
Sensitivity analysis of the interaction between exposure to early-life famine and Dietary Inflammatory Index on the incidence of hypertension. DII, dietary inflammatory index; the Dietary Inflammatory Index is calculated based on food nutrient components and nutrients derived from dietary surveys; In this sensitivity analysis, the mean DII was calculated using only dietary assessments collected prior to the first incident hypertension event for each participant. DII Q2, DII Q3, and DII Q4 represent the second, third, and fourth quartiles (Q2, Q3, Q4) of the Dietary Inflammatory Index, respectively, with Q1 as the reference group; CI: confidence interval; *Compared with the reference group, *P* < 0.05; Adjusted for age, residence area, gender, educational level, BMI, smoking status, alcohol consumption, physical activity level, and mean daily intakes of energy over 3 days.

The mean DII values from 2004 to 2011 were categorized into quartiles and analyzed using Cox proportional hazards regression models. The quartile cut-points were −1.74, −0.67, and 0.43. The results showed that participants in the third DII quartile (Q3) had a significantly increased risk of incident hypertension (HR = 1.39, 95% CI: 1.05–1.83), indicating that a moderate-to-high dietary inflammatory potential may increase the risk of hypertension. Further analysis examining the interaction between early-life famine exposure and DII revealed a significant interaction effect in the highest DII quartile (Q4) for the early childhood exposure group (HR = 1.79, 95% CI: 1.01–3.20), indicating that individuals with both early childhood famine exposure and highly pro-inflammatory diets faced the greatest hypertension risk. No significant interactions were observed in other DII quartiles. Detailed results are presented in Figure 3. In a sensitivity analysis, mean DII was recalculated using only dietary assessments collected prior to the first incident hypertension event and was not updated thereafter. The overall pattern remained broadly similar to that of the primary analysis. Although the significant overall association shifted from Q3 to Q4, statistically significant excess risk was still concentrated in the higher DII quartiles, supporting the robustness of the observed trend. Detailed results are shown in Figure 4.

## 4 Discussion

This study utilized the CHNS, a nationwide, large-scale, long-term longitudinal cohort study in China, to investigate the incidence of hypertension among Chinese adults born in the 1950s−1960s, based on approximately 12 years of follow-up. The results showed that famine exposure during early childhood was significantly associated with an increased risk of hypertension onset in adulthood. By introducing DII as a categorical variable and analyzing its interaction with early-life famine exposure, we suggest that dietary pro-inflammatory potential modifies the association between early childhood famine exposure and hypertension risk in adulthood. This study offers insights into the public health implications of hypertension prevention in China. Targeted anti-inflammatory dietary interventions for high-risk populations may offer new perspectives for hypertension prevention and the management of chronic diseases.

Our results are broadly consistent with previous studies, supporting the observed association between early-life nutritional status and adult cardiovascular risk. In hypertension risk studies, Ogah et al. (42) reported a significantly increased risk (OR = 2.47, 95% CI: 1.14–5.36) among Biafran famine survivors exposed at ages 1–5. Similarly, data from the China Health and Retirement Longitudinal Study (41) indicated that childhood exposure to the Great Famine was associated with a higher risk of hypertension in middle age (OR = 1.64, 95% CI: 1.44–1.87). Currently, there are relatively few studies focusing on incident hypertension. Using data from the Kailuan cohort, Jiao (43) found that both fetal and early childhood famine exposure increased the risk of hypertension in adulthood and blood pressure outcomes in adulthood compared to the non-exposure group. Some scholars attribute this increased risk to epigenetics and metabolic reprogramming mechanisms. Early-life malnutrition may disrupt DNA methylation and the hypothalamic-pituitary-adrenal axis, thereby altering gene expression and impairing physiological homeostasis and developmental maturation. These changes ultimately increase susceptibility to adult hypertension (44, 45). In this study, the association between fetal-period famine exposure and incident hypertension was not significant, whereas a similar association has been observed in other studies that utilized the Great Chinese Famine (46). Potential differences in study design, such as the definition of fetal exposure, the selection of covariates, and participant recruitment criteria, may have accounted for the non-significant association observed in our study (42).

In the present study, a higher DII was associated with an increased risk of incident hypertension. Consistent with the findings of this study, multiple studies have reported a significant association between dietary pro-inflammatory effects and hypertension prevalence in the higher DII quartiles (47–49). In studies on hypertension risk, Dong et al. (19) found that participants in the DII Q3 and Q4 groups had a significantly increased risk of hypertension after adjusting for covariates (Q3: OR = 1.14, 95% CI: 1.05–1.23; Q4: OR = 1.17, 95% CI: 1.07–1.26). In studies on the risk of incident hypertension, Xu et al. (18) found that DII Q4 was significantly associated with incident hypertension (HR = 1.13, 95% CI: 1.02–1.24), while the association between DII Q3 and incident hypertension was not significant. This association may be closely related to the inflammatory potential of the diet. Specifically, higher DII scores are linked to elevated inflammatory markers (e.g., CRP, TNF-α, and IL-1) (50, 51), which promote systemic inflammation, oxidative stress, and endothelial dysfunction, thereby contributing to hypertension pathogenesis (52).

More importantly, we observed a significant interaction between early-life famine exposure and DII, suggesting that DII may modify the association between early-life famine exposure and incident hypertension. This observed interaction can be partially explained by the early-life “DOHaD” theory and the thrifty phenotype hypothesis. During infancy, children's nutritional needs gradually increase, and the introduction of complementary foods exposes them to a wider variety of foods, supporting physical and cognitive development and leading to improvements in motor coordination and cognitive behavior (53, 54). It is precisely during this critical period of rapid growth that adverse factors, such as malnutrition, may influence the body's internal environmental homeostasis through epigenetic mechanisms, chronic renal developmental changes, and endocrine axis secretion disorders, resulting in long-term effects on the metabolic system and increased susceptibility to inflammation, as well as impaired blood pressure regulation (55, 56). No significant interaction was observed for fetal famine exposure, which may be partly related to less precise exposure classification and postnatal nutritional or environmental compensation that attenuated the association.

This study has several advantages. First, it used the CHNS, a nationally representative longitudinal cohort with long follow-up and rigorous data collection, supporting the robustness and generalizability of our findings. With birthplace information available, we were able to assess the severity of famine during early life. Second, this study incorporated DII as a categorical variable into interaction analysis, systematically exploring the synergistic effects of different famine exposure periods and dietary pro-inflammatory effects on the incidence of hypertension. Third, the DII was derived from repeated dietary assessments across multiple survey waves, which may better capture the long-term inflammatory potential of diet than a single dietary measurement. Furthermore, sensitivity analyses using an alternative pre-event DII definition and further adjustment for sodium and potassium intake yielded broadly similar results, further supporting the robustness of the findings.

Several limitations should be acknowledged. First, due to the limitations of the CHNS data, the absence of precise birth-month information may lead to misclassification of the fetal-exposure group. Second, because exposure groups are defined by birth cohorts, baseline age was structurally linked to the exposure classification; therefore, cohort- and age-related residual confounding cannot be fully excluded. Despite these limitations, using a clearly defined historical window and birth-cohort-based exposure classification has been widely adopted in this field, offering a unique opportunity to explore DOHaD-related hypotheses. Third, dietary data were also only available through 2011, potentially limiting the assessment of diet closer to the end of follow-up. Finally, although we adjusted for multiple covariates and used regional EMR to characterize geographic differences in famine severity, residual confounding may remain because individual-level famine experiences, socioeconomic and community-level conditions, and birth-cohort-related life-course changes could not be fully captured.

## 5 Conclusions

In summary, this study suggests that, among Chinese adults, exposure to famine during early childhood may modify the association between a more pro-inflammatory diet and incident hypertension in adulthood. These findings may have public health implications for life-course hypertension prevention, supporting targeted blood pressure monitoring and the promotion of less pro-inflammatory dietary patterns among Chinese adults with early-life nutritional adversity. Sustained nutritional support and anti-inflammatory dietary guidance for vulnerable populations may also help reduce the long-term burden of non-communicable diseases. Future studies incorporating more precise birth information and regional famine intensity indicators could further validate and refine these findings.

## Data Availability

The datasets presented in this study can be found in online repositories. The names of the repository/repositories and accession number(s) can be found below: https://www.cpc.unc.edu/projects/china.
