# Supplementary material for: The association between early-life famine exposure and hypertension risk in adulthood and its interaction with dietary inflammatory index among Chinese adults
Source: Front Nutr. 2026 Jun 17;13:1841432. doi: 10.3389/fnut.2026.1841432 (PMC13322136; doi:10.3389/fnut.2026.1841432)
Supplement: Supplementary file 1 [file Table_1.docx]

**Table S1.** Sensitivity analysis of famine exposure and risk of incident hypertension after adjusting for dietary sodium and potassium intake

|  | **Incidence (%)** | **Model 1** | | **Model 2** | | **Model 3** | |
| --- | --- | --- | --- | --- | --- | --- | --- |
|  |  | **HR (95% CI)** | ***P*** | **HR (95% CI)** | ***P*** | **HR (95% CI)** | ***P*** |
| Non-exposure group | 33.5 | Ref. |  | Ref. |  | Ref. |  |
| Fetal exposure group | 35.9 | 1.07 (0.87–1.32) | 0.504 | 1.07 (0.87–1.32) | 0.510 | 1.05 (0.85–1.30) | 0.651 |
| Early childhood  exposure group | 39.6 | 1.22 (1.02–1.46)* | 0.032* | 1.21 (1.02–1.45) | 0.039* | 1.22 (1.01–1.46)* | 0.035* |

Note: CI: confidence interval; Model 1 included gender as an adjustment variable; Model 2 included gender, residence area, educational level, and BMI as adjustment variables; Model 3 included gender, residence area, educational level, BMI, smoking status, alcohol consumption, physical activity level, and mean daily intakes of energy, sodium and potassium over three days as adjustment variables; *Compared with the non-exposure group, *P* < 0.05.

**Table S2.** Dietary Inflammatory Index (DII) across famine-exposure birth cohorts [*P*_50_ (*P*_25_, *P*_75_)]

|  | **Non-exposure group** | **Fetal exposure group** | **Early childhood**  **exposure group** |
| --- | --- | --- | --- |
| DII 2004 | -1.07 (-1.97, 0.02) | -1.15 (-1.90, 0.04) | -1.09 (-2.06, 0.02) |
| DII 2006 | -0.96 (-1.88, -0.02) | -0.94 (-1.88, 0.36) | -0.64 (-1.74, 0.45)* |
| DII 2009 | -1.05 (-1.90, 0.05) | -1.04 (-1.99, 0.07) | -1.04 (-1.96, 0.04) |
| DII 2011 | 0.97 (0.14, 1.84) | 1.13 (0.30, 2.02) | 1.21 (0.14, 1.95) |
| DII 2004–2011 | -0.55 (-1.17, 0.18) | -0.47 (-1.20, 0.30) | -0.41 (-1.17, 0.29) |

Note: Data are expressed as *P*_50_ (*P*_25_, *P*_75_); DII: Dietary Inflammatory Index; DII 2004, DII 2006, DII 2009, and DII 2011 represent the Dietary Inflammatory Index for the years 2004, 2006, 2009, and 2011, respectively; The Dietary Inflammatory Index is calculated based on the food nutrient components and nutrients derived from dietary surveys; DII 2004–2011 represents the average Dietary Inflammatory Index from 2004 to 2011. Intergroup comparisons were performed using the nonparametric Kruskal-Wallis test. Pairwise comparisons were conducted using the Wilcoxon rank-sum test with Benjamini-Hochberg adjustment. *Compared with the non-exposure group, *P* < 0.05.
